# Establishment of a duplex real-time qPCR method for detection of *Salmonella* spp. and *Serratia fonticola* in fishmeal

**DOI:** 10.1186/s13568-020-01144-x

**Published:** 2020-11-24

**Authors:** Jinghua Ruan, Wujun Wang, Tiyin Zhang, Teng Zheng, Jing Zheng, Shiyu Yu, Daojin Yu, Yifan Huang

**Affiliations:** 1 College of Life Sciences, Fujian Agriculture and Forestry University, Fuzhou, 350002 Fujian People’s Republic of China; 2 Fujian Key Laboratory of Traditional Chinese Veterinary Medicine and Animal Health, Fujian Agriculture and Forestry University, Fuzhou, 350002 Fujian People’s Republic of China; 3 Fujian Key Laboratory for Technology Research of Inspection and Quarantine, Technology Center of Fuzhou Customs District, Fuzhou, 350002 Fujian People’s Republic of China; 4 College of Animal Science, Zhejiang University, Hangzhou, 310058 Zhejiang People’s Republic of China

**Keywords:** *Salmonella* spp., *S. fonticola*, Duplex real-time qPCR, Feed safety

## Abstract

*Salmonella* spp. is a high-risk bacterial pathogen that is monitored in imported animal-derived feedstuffs. *Serratia fonticola* is the bacterial species most frequently confused with *Salmonella* spp. in traditional identification methods based on biochemical characteristics, which are time-consuming and labor-intensive, and thus unsuitable for daily inspection and quarantine work. In this study, we established a duplex real-time qPCR method with *invA*- and *gyrB*-specific primers and probes corresponding to *Salmonella* spp. and *S. fonticola*. The method could simultaneously detect both pathogens in imported feedstuffs, with a minimum limit of detection for *Salmonella* spp*.* and *S. fonticola* of 197 copies/μL and 145 copies/μL, respectively (correlation coefficient R^2^ = 0.999 in both cases). The amplification efficiency for *Salmonella* spp. and *S. fonticola* was 98.346% and 96.49%, respectively. Detection of fishmeal was consistent with method GB/T 13091-2018, and all seven artificially contaminated imported feed samples were positively identified. Thus, the developed duplex real-time qPCR assay displays high specificity and sensitivity, and can be used for the rapid and accurate detection of genomic DNA from *Salmonella* spp. and *S. fonticola* within hours. This represents a significant improvement in the efficiency of detection of both pathogens in imported feedstuffs.

## Introduction

*Salmonella* spp. are ubiquitous Gram-negative bacteria in the environment and include six different subspecies and more than 2000 serotypes that infect a wide range of hosts, often causing severe food poisoning outbreaks in humans and other animals. People infected with *Salmonella* can develop diarrhea, fever, and suffer dehydration, hence *Salmonella* spp. are of significance to public health.

*S. fonticola* is a species belonging to the *Serratia* genus that was first isolated from water and soil in 1979 (Gavini et al. 1979). Subsequent studies showed that *S. fonticola*, a member the Gram-negative *Enterobacteriaceae* family that includes *Salmonella* spp., is also ubiquitous in environments such as water, soil, plants, and the gastrointestinal tract of humans and other animals (Tasic et al. 2013). Research has revealed that *S. fonticola* can infect various tissues and organs in humans, causing septic arthritis, septicemia, gastrointestinal tract infections, and surgical infections (Rd et al. 1985; Bollet et al. 1991; Kunimoto et al. 2004). Therefore *S. fonticola* have been defined as an important opportunistic pathogen.

At present, the detection of *Salmonella* spp. in imported animal-derived feeds (fish meal and chicken powder) involves non-selective enrichment, selective enrichment, selective platelet culturing, biochemical culturing of suspected *Salmonella* colonies (triglyceride tests, etc.), and even serological typing. These conventional methods require at least 3 days to detect *Salmonella* spp. in daily quarantine work. Furthermore, suspected *Salmonella* colonies based on selective plate isolation and culturing of imported animal-derived feeds often turn out to be *S. fonticola* when biochemical properties are investigated (Chen et al. 2016). The enzyme linked immune sorbent assay (ELISA) method is commonly used for detecting pathogens in imported animal-derived feeds, but this method can produce incorrect results due to its cumbersome operational steps. A novel method involving polymer fluorescent nanoparticles as biosensor probes has been described for the detection of *Salmonella* (Jain et al. 2016), which takes only 3 h, but cannot be applied to large volume detection due to its prohibitively high cost.

Traditional PCR and multiplex PCR methods have been developed for the detection of *Salmonella* species and *Serratia* genus attributed to its high sensitivity, specificity, and results can be obtained within several hours. Multi-PCR approaches can differentiate two or more pathogens in one amplification, however, products are easily contaminated during agarose gel electrophoresis, which increases false-positive and false-negative results. Thus, a more reliable approach such as real-time fluorescence quantitative PCR (RT-qPCR) would be more desirable for the rapid and accurate detection of pathogens. RT-qPCR methods are known to be fast, reliable, and highly efficient. Several single RT-qPCR methods for detecting *S. nematodiphila* (Hurst et al. 2008), *S. marcescens* (Iwaya et al. 2005; Joyner et al. 2014; Cornegliani et al. 2015) and *Salmonella* spp. (Perelle et al. 2004; Nam et al. 2005; Tomás et al. 2009) have been described.

Previous studies on fish meal and other animal-derived feeds have mainly focused on the detection and epidemiology of *Salmonella*, *Shigella*, *Escherichia coli*, and other common pathogenic bacteria. A method for the simultaneous detection of *Salmonella* spp. and *S. fonticola* in imported animal-derived feeds using RT-qPCR has not been reported. Such a method could provide rapid differential diagnosis of suspected *Salmonella* colonies after selective plate separation and culturing. This would undoubtedly improve the detection of *Salmonella*, and greatly shorten the time required for subsequent biochemical and serological identification, saving valuable manpower and material resources.

The duplex real-time qPCR method established in the present work provides a useful tool for the simultaneous detection of *Salmonella* spp. and *S. fonticola*. The method has important theoretical significance and great potential for improving the safety of imported feeds by rapidly identifying bacterial pathogens and facilitating effective quarantining in a more timely manner than traditional detection methods.

## Materials and methods

### Bacterial strains

A total of 48 tested strains were used in this study, including 17 reference strains from six different collection centers, and 31 isolates from imported fishmeal (Additional file 1: Tables S1–S3). All experimental strains were streaked on nutrient agar plates and cultured in Luria–Bertani (LB) broth at 37 °C overnight (~ 18–24 h), except for *S. marcescens*, which was grown at 23 °C for 24 h. An established single colony was inoculated into 3 mL of LB broth for 8 h and the resultant culture was harvested for genomic DNA extraction.

### Sample collection and bacterial isolation

A 25 g sample of fishmeal was added to 225 mL of buffered peptone water (BPW) and incubated at 36 ± 1 °C for between 16 − 20 h. A 10 mL sample of this pre-enrichment culture was transferred into 100 mL of enrichment solution containing selenite cysteine (SC enrichment solution), cultured at 36 ± 1 °C for 24 h, and inoculated onto CHROMagar™ Salmonellae (CHROMagar) designed for *Salmonella* spp. Two *Salmonella* spp. colonies were cultured at 36 ± 1 °C for 48 h in CHROMagar, one on the medium slope, the other punctured through the agar, and both were then inoculated on trisaccharide iron medium. Meanwhile, suspicious colonies were inoculated onto lysine decarboxylase medium and cultured at 36 ± 1 °C for 18 − 24 h (or up to 48 h if necessary). Colonies from positive samples presumed to be *Salmonella* spp. were further identified using a VITEK II Compact 30 instrument (BioMérieux) with a GN card according to the manufacturer’s specifications. DNA extraction from isolates and reference strains was performed with a TIANamp Bacteria DNA Kit (Tiangen Biotech, Co., Ltd, Beijing, China) according to the manufacturer’s recommendations.

### Species-specific primers and probes design

The *invA* sequences of *Salmonella* spp. were aligned to identify conserved and specific regions using CLUSTAL W software (Aiyar 2000). A series of sense and antisense primers were designed based on these conserved and specific regions using ABI ViiA7 PrimerExpress software, and the final specific primer pair and dual-labelled probe (Additional file 1: Table S4) targeting the *Salmonella* spp. virulence gene were determined using the basic logical alignment search tool (BLAST) (Altschul et al. 1997). Primers and dual-labelled probe targeting the *gyrB* gene were as described our previous research (Ruan et al. 2017). The primers and probes sets were synthesized by Sangon Biotech (Shanghai, China), and probes labeled with the fluorescent reporter dye carboxy-4′,5′-dichloro-2′,7′-dimethoxyfluorescein (JOE) targeting *invA* and 6-carboxyfluorescein (FAM) targeting *gyrB* were covalently coupled to the 5′-end, with Black Hole Quencher 1 (BHQ-1) at the 3′-end.

### Single real-time qPCR assays

The optimum annealing temperature of primers were screened using a Bio-rad CFX96 Real-time PCR system (Bio-Rad Laboratories, Inc., USA). Two simplex RT-qPCR detection methods for the detection of *Salmonella* spp. and *S. fonticola* were established using primers and probes targeting *invA* and *gyrB* genes, respectively, using an Applied Biosystems ViiA 7 real-time PCR system (Life Technologies Inc., Foster City, CA, USA). Reaction conditions were determined after various PCR parameters were tested according to the information supplied with the reagents. The optimized 20 μL PCR contained 10 μL of 2 × Premix Ex Taq (TaKaRa Biomedical Technology Co., Ltd), 0.4 μL of each primer (10 μM) and probe (10 μM), 0.2 μL of ROX Reference Dye (50 ×), 1 μL of bacterial DNA, and 7.6 μL of Rnase-free ddH_2_O. Optimized thermal cycling conditions involved an initial denaturation at 95 °C for 30 s, followed by 40 cycles at 95 °C for 5 s and 64 °C for 34 s. ViiA 7 Software was employed to monitor PCR amplification, and the collect and analyze amplification data.

To verify the specificity of the two simplex RT-qPCR assays, tests were performed by amplifying genomic DNA extracted from strains. We constructed recombinant plasmids carrying *invA* and *gyrB* gene to determine the sensitivity of two simplex real-time qPCR. The standard plasmids of pMD-*invA* and pMD-*gyrB* were serially diluted 9 times with ten-fold and then subjected to RT-qPCR to make standard curves(three technical replications for each dilution), from which we determined both the amplification efficiency and the minimum detection limit of the two real-time qPCR methods. The plasmid copy numbers was calculated using the following formula (Whelan et al. 2003):

$$ {\text{Copy number }} = \, \left( {{\text{DNA amount }}\left( {{\text{ng}}} \right) \, \times { 6}.0{22} \times {1}0^{{{23}}} /{\text{length }}\left( {{\text{bp}}} \right) \, \times { 1}0^{{9}} \times { 65}0} \right). $$

### Duplex real-time qPCR assay

A duplex real-time qPCR assay was established based on the two simplex RT-qPCR assays developed for the detection of *Salmonella* spp. and *S. fonticola* using an equal amount of genomic DNA from the two pathogens in the same reaction. Optimal PCR conditions were ultimately determined based on the simplex RT-qPCR parameters described above by varying a single factor while all other parameters remained constant. The main factor to be investigated was the concentration of the two primer and probe sets since the annealing temperatures of primers and probes was already optimized.

To analyze the reproducibility and stability of the established duplex real-time qPCR method, recombinant pMD-*invA* and pMD-*gyrB* plasmids were tenfold serially diluted five times (each dilution was tested in triplicate). A total of 96 reactions were simultaneously performed using the *invA*- and *gyrB* recombinant plasmids in the same real-time qPCR mixture to verify reproducibility. The coefficient of variation (CV) based on quantification cycle (Cq) values for each test was used to evaluate the performance of this approach.

### Detection of artificially contaminated feed stuffs

A 10 mL sample of this pre-enrichment culture was transferred into 100 mL of SC enrichment solution, cultured at 36 ± 1 °C for 24 h, and inoculated onto CHROMagar designed for *Salmonella* spp. Two *Salmonella* spp. colonies were cultured at 36 ± 1 °C for 48 h in CHROMagar, one on the medium slope, the other punctured through the agar, and both were then inoculated on trisaccharide iron medium. Meanwhile, suspicious colonies were inoculated onto lysine decarboxylase medium and cultured at 36 ± 1 °C for 18 − 24 h (or up to 48 h if necessary).

We tested seven imported fishmeal samples not contaminated by either *Salmonella* spp. or *S. fonticola*. A 25 g portion of three of the fishmeal samples was artificially contaminated with *Salmonella enteritidis* and *S. fonticola* (2 × 10^6^ CFU/g), while the other four samples were contaminated with *Salmonella enteritidis* (2 × 10^6^ CFU/g). Contaminated samples were added to 225 mL of BPW enrichment solution and incubated at 36 ± 1 °C for 16 − 20 h. A 1 mL sample of the pre-enrichment culture was transferred into 10 mL of SC enrichment solution, cultured at 36 ± 1 °C for 24 h. Then 1 mL of above solution was used for genomic DNA extraction using the TIANamp Bacteria DNA Kit (Tiangen Biotech, Co., Ltd). Finally, RT-qPCR was performed for the detection of *Salmonella* spp. and *S. fonticola*, and the national standard method (GB/T13091-2018) was also employed for verification.

## Results

### Determination of optimum annealing temperature

Genomic DNA from *Salmonella enteritidis* and *S. fonticola* was used as a template to determine optimal annealing temperatures for the two primer sets by testing between 55 and 65 °C using a Bio-Rad fluorescence quantitative PCR instrument. RT-qPCR using the designed primers and probes yielded the highest fluorescence intensity of amplified products at an annealing temperature of 64.5 °C for both *invA-* and *gyrB-* specific primers and probes, and this annealing temperature was employed in subsequent experiments.

### Specificity of the single real-time qPCR assay

The specificity of the two single real-time qPCR assays was verified by amplifying genomic DNA extracted from reference strains and laboratory isolates. The real-time qPCR assay detected *Salmonella* spp. only, while no fluorescent signal was observed for any non-*Salmonella* spp. strains or blank controls. Additionally, the real-time qPCR assay targeting *S. fonticola* generated four specific amplification curves for the detection of four *S. fonticola* strains, while non-*S. fonticola* and blank controls were not amplified. Thus, the specificity of the two real-time qPCR assays was 100%, with no detectable fluorescent signal for negative samples or blank controls.

### Standard curves and sensitivity of the simplex real-time qPCR assay

**Fig. 1:**
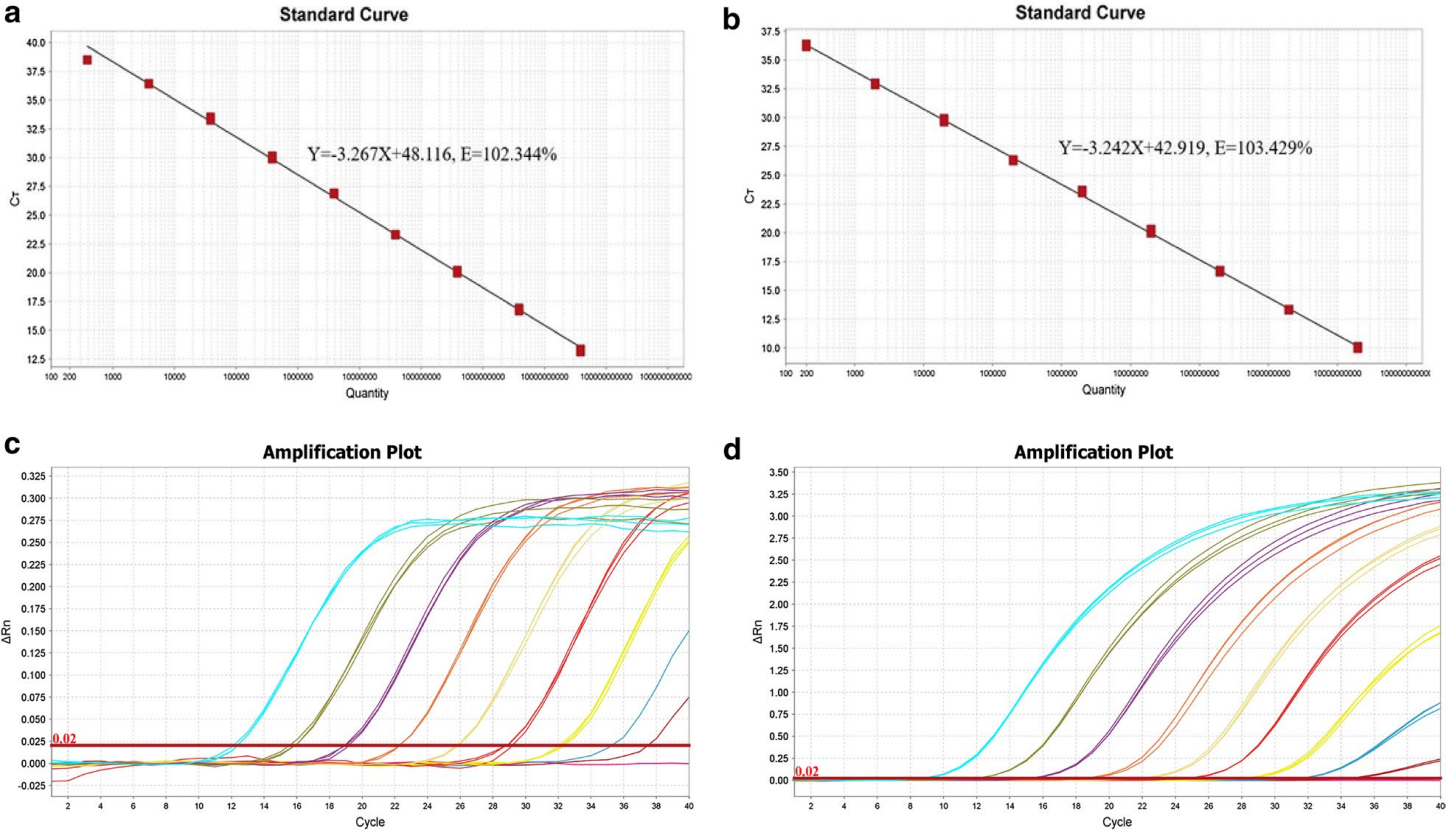
Standard curves and sensitivity of the real-time qPCR assay. Standard curves for *Salmonella*. app (**a**) and *S. fonticola* (**b**) RT-qPCR assay. The curves represent a range from 10^10^ to 10^2^ copies per reaction. Sensitivity of the real-time qPCR assay for the detection of *Salmonella* species (**c**) and *S. fonticola* (**d**). Amplification plots from left to right represent a range of *invA*/*gyrB* gene-containing plasmids from 1.97 × 10^10^ to 1.97 × 10^2^ copies/μL (1.45 × 10^10^ to 1.45 × 10^2^ copies/μL), respectively

Recombinant *invA*- and *gyrB*-containing plasmids were tenfold serially diluted nine times, resulting in real-time qPCR amplicon copy numbers from 1. 97 × 10^10^ copies/μL to 1. 97 × 10^2^ copies/μL, and 1.45 × 10^10^ copies/μL to 1.45 × 10^2^ copies/μL, respectively. The standard curve for *invA* has a γ intercept of 48.116, a slope of -3.267, and a mean efficiency of 102.344% (Fig. 1a). The *gyrB* standard curve has a γ intercept of 42.919, a slope of -3.242, and a mean efficiency of 103.429% (Fig. 1b). As shown in Fig. 1c, d, the single quantitative real-time PCR assays could detect *Salmonella* spp. and *S. fonticola* at concentrations as low as 197 and 145 copies per reaction, respectively.

### Establishment of the duplex real-time qPCR

**Fig. 2:**
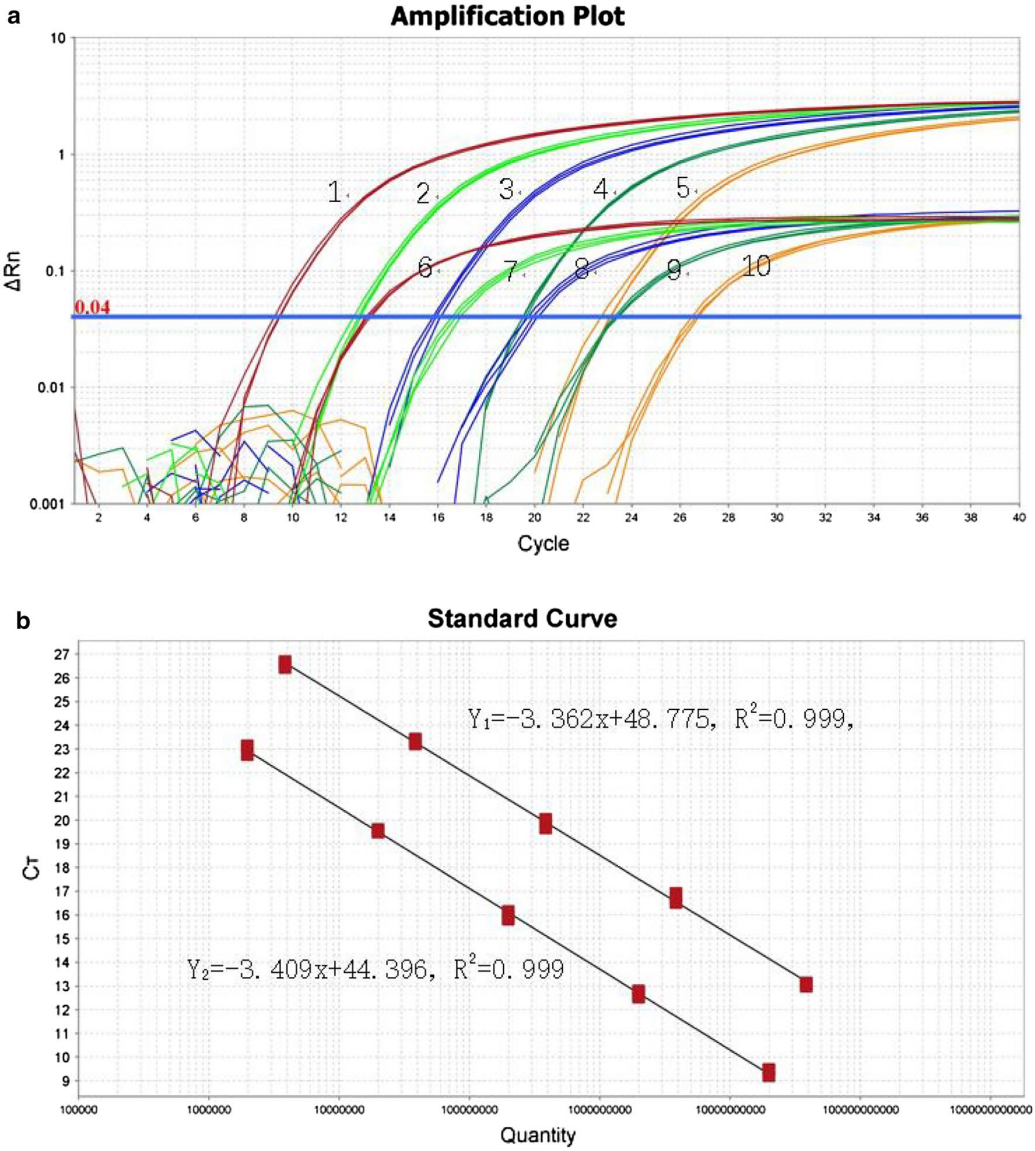
Amplification plot for the duplex RT-qPCR assay using serially diluted plasmids (**a**). Amplification plots 1–5 represent *gyrB* gene-containing plasmid ranging from 1.97 × 10^10^ to 1.97 × 10^6^ copies/μL, respectively. Amplification plots 6–10 represent *invA* gene-containing plasmid ranging from 1.45 × 10^10^ to 1.45 × 10^6^ copies/μL, respectively. Standard curves for the duplex RT-qPCR assay (**b**). Y_1_ and Y_2_ are standard curves using *invA* gene*-* and *gyrB* gene-containing plasmids, respectively

Figure 2a, b shows amplification plots and standard curves for the duplex real-time qPCR assay established for simultaneous detection of *Salmonella spp.* and *S. fonticola* developed with recombinant pUCm-*invA* and pUCm-*gyrB* plasmids. Standard curve slopes are − 3.362 and − 3.409 for the detection of *invA* and *gyrB*, respectively, indicating an amplification efficiency of 98.346% and 96.49%. A correlation coefficient consistently higher than 0.999 indicates effective simultaneous detection of two kinds of pathogens in one real-time qPCR assay without cross-reaction.

### Tests of reproducibility and stability

Five recombinant *invA* and *gyrB* plasmid dilutions were simultaneously used as substrates to evaluate the reproducibility and stability of the assay. As shown in Additional file 1: Table S5, the standard deviation was no more than 0.153 for all reactions, and the coefficient of variation was less than 0.73%. Additionally, Ct values for the same plasmid samples obtained from same experiments were determined to analyze the performance of the developed real-time qPCR method. The results showed that amplification plots for all 96 replicate reactions were almost coincident in the vicinity of the threshold line (Additional file 2: Fig. S1). Thus, the established quantitative real-time PCR assay has high stability and reproducibility.

### Duplex real-time qPCR analysis of imported feed stuffs

**Fig. 3:**
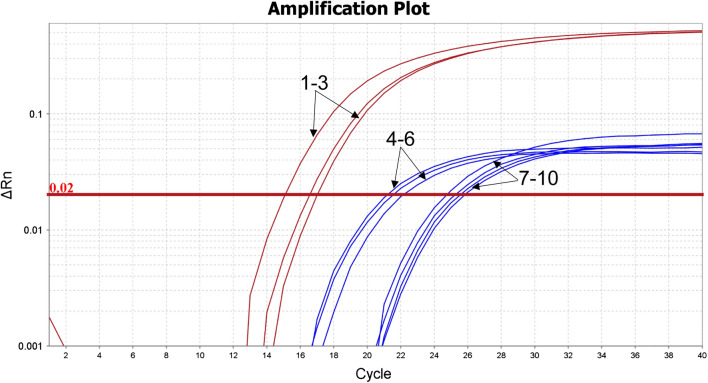
Detected amplification plot of artificially contaminated Feedstuffs. Red curves 1–3 and blue curves 4–6 represent the amplification plots of *gyrB* and *invA*-specific primers, respectively, which correspond to three samples artificially contaminated with *Salmonella enteritidis* and *S. fonticola*. Blue curves 7–10 represent the amplification plots of *invA*-specific primers, which correspond to four samples artificially contaminated with *Salmonella enteritidis* only

Imported feedstuffs not infected with either *Salmonella* spp. or *S. fonticola* were artificially contaminated with the corresponding pathogens. Three feed samples were contaminated with *Salmonella enteritidis* and *S. fonticola*, while four samples were contaminated with *Salmonella enteritidis* only. Expected fluorescent signals were observed among all artificially contaminated feed samples by the end of amplification (Fig. 3). Meanwhile, the results of the detection of artificial contaminated samples were consistent with the national standard method (GB/T13091-2018) that was also employed for verification.

## Discussion

Many target genes for the detection of *Salmonella* genus have been reported, including *afgA*, *hilA*, *spvC*, *sef* (Crăciunaş et al. 2012; Mann et al. 2013), *fliC*, *fliB*, *iroB*, *rfbJ *(Shanmugasundaram et al. 2009), *ompC* (Ngan et al. 2010), *spvR* (Mahon et al. 1993), *fimA *(Cano et al. 1993), *viaB* (Hashimoto et al. 1995),etc. However, they have shortcomings for the identification of *Salmonella* species, and false-positives limit the diagnostic process. At present, quorum sensing-related such as *luxS* and *gyrB* are widely used, along with virulence genes *invH*, *sopE*, *hilA*, and *invA* in the SP11, *sugR*, *rhuM*, and *iacP* in the SPI-3 pathogenicity island, and *spvB* and *spvC* in virulence plasmids. Of these, *invA*, which encodes an epithelial cell surface protein, is present in all *Salmonella* species, and is the most widely reported target gene in the *Salmonella* genus. Primers and probes for the diagnosis of *Salmonella* spp. based on this conserved gene are highly specific, hence *invA* was selected as the target gene for real-time qPCR detection of *Salmonella* species in the present study.

The 16S rDNA gene has been used for classification and identification of *Serratia* species in previous studies, as has the quorum sensing gene *luxS* (Zhu et al. 2008), which was employed in real-time qPCR (Joyner et al. 2014). In addition, several studies used carbapenem antibiotic resistance genes for identification of *Serratia* species (Yamamoto and Harayama 1996). The *gyrB* gene, encoding the B subunit of DNA gyrase (GyrB) that forms topoisomerase II, an essential protein in replication, transcription, DNA synthesis, and maintenance of the DNA supercoiled structure, is more reliable than the 16S rDNA gene for the identification of bacterial species (Gellert et al. 1976). The protein-coding *gyrB* gene contains more genetic information than the non-protein-coding 16S rDNA, resulting in a greater capacity to distinguish bacterial species (Kasai et al. 1998). Thus, the *gyrB* gene was selected for discriminating *S. fonticola* and *Salmonella* spp. using real-time qPCR.

Real-time qPCR requires stricter primers, amplification products, and reaction conditions than conventional PCR. Primers used for real-time qPCR are typically 18 − 30 bp in length. Shorter primers (< 15 nucleotides) can be combined efficiently, but often to the detriment of specificity. By contrast, longer primers often display enhanced specificity but may also hybridize with the wrong pairing sequence, reducing specificity and decreasing the efficiency of hybridization, resulting in diminished PCR amplification. Ideal results are generally obtained when amplifying products of less than 300 bp. Therefore, the PCR products selected for differentiating *Salmonella* spp. and *S. fonticola* were 199 bp and 94 bp, respectively. Different annealing temperatures were tested to determine the optimum annealing temperature of the two pairs of primers, in order to reduce the influence of annealing temperature on the duplex real-time qPCR experiment. An annealing temperature of 64 °C was found to be optimal.

The duplex real-time qPCR approach established for the rapid detection of *Salmonella* spp. and *S. fonticola* in imported feedstuffs was characterized by a high correlation coefficient between the Ct value and the logarithm of the initial copy number (R^2^ = 0.999). Additionally, parameters including slope, y-axis intercept, and amplification efficiency between duplex real-time PCR and single real-time PCR were compared. As shown in Additional file 1: Table S6, the slope of the standard curve and its intercept on the y-axis are approximately equal, and the amplification efficiency is ~ 100%. Thus, simultaneous detection of the two pathogens was achieved in a single duplex real-time qPCR amplification assay, and the detection limit of this method is suitable for daily inspection and quarantine work. In summary, the rapid diagnostic method established in this study has many advantages, including a low detection limit and high repeatability. It is also rapid and convenient to deploy, since the results can be obtained within several hours after pre-enriching, representing a significant improvement in efficiency for detecting *Salmonella* spp. in imported animal-derived feedstuffs during quarantine work. This method is of great theoretical and practical value for ensuring the safety of imported feedstuffs.

## Supplementary information

**Additional file 1:** **Table S1.*** Salmonella* spp. strains used in this study. **Table S2.*** Serratia* genus strains used in this study. **Table S3.** Other strains used in this study. **Table S4.** Real-time qPCR primer pairs and probes used in this study. **Table S5.** Reproducibility and stability test of the duplex real-time PCR. **Table S6.** Parameters comparison between duplex real-time PCR and single real-time PCR.

**Additional file 2: Fig. S1.** Repeatability and stability test of RT-qPCR. 96 amplification plots for detection of *Salmonella enteritidis* (A) and *S. fonticola* (B).

## Data Availability

All data generated and analyzed during this study are included in this published article.

## References

[CR1] Aiyar A (2000). The use of CLUSTAL W and CLUSTAL X for multiple sequence alignment. Meth Mol Biol.

[CR2] Altschul SF, Madden TL, Schäffer AA, Zhang J, Zhang Z, Miller W, Lipman DJ (1997). Gapped BLAST and PSI-BLAST: a new generation of protein database search programs. Nucleic Acids Res.

[CR3] Bollet C, Gainnier M, Sainty JM, Orhesser P, Micco PD (1991). *Serratia fonticola* isolated from a leg abscess. J Clin Microbiol.

[CR4] Cano RJ, Rasmussen SR, Sánchez FG, Palomares JC (1993). Fluorescent detection-polymerase chain reaction (FD-PCR) assay on microwell plates as a screening test for *salmonellas* in foods. J Appl Bacteriol.

[CR5] Chen Y, Wu D, Sun M, Deng M, Cui S, Liang C, Geng J, Sun T, Long L, Xiao X (2016). Serum bactericidal assay: new role in *Salmonella* detection. J Aoac Int.

[CR6] Cornegliani L, Corona A, Vercelli A, Roccabianca P (2015). Identification by real-time PCR with SYBR Green of *Leishmania* spp. and *Serratia marcescens* in canine ‘sterile’ cutaneous nodular lesions. Vet Dermatol.

[CR7] Crăciunaş C, Keul AL, Flonta M, Cristea M (2012). DNA-based diagnostic tests for Salmonella strains targeting hilA, agfA, spvC and sef genes. J Environ Manage.

[CR8] Gavini F, Ferragut C, Izard D, Trinel PA, Leclerc H, Lefebvre B, Mossel DAA (1979). *Serratia fonticola*, a new species from water. I J Syst Bacteriol.

[CR9] Gellert M, Mizuuchi K, O'Dea MH, Nash HA (1976). DNA gyrase: an enzyme that introduces superhelical turns into DNA. Proc Natl Acad Sci U S A.

[CR10] Hashimoto Y, Itho Y, Fujinaga Y, Khan AQ, Sultana F, Miyake M, Hirose K, Yamamoto H, Ezaki T (1995). Development of nested PCR based on the *ViaB* sequence to detect *Salmonella typhi*. J Clin Microbiol.

[CR11] Hurst MRH, Young SD, O'Callaghan M (2008). Development of a species-specific probe for detection of *Serratia entomophila* in soil. N Z Plant Prot.

[CR12] Iwaya A, Nakagawa S, Iwakura N, Taneike I, Kurihara M, Kuwano T, Gondaira F, Endo M, Hatakeyama K, Yamamoto T (2005). Rapid and quantitative detection of blood *Serratia marcescens* by a real-time PCR assay: its clinical application and evaluation in a mouse infection model. Fems Microbiol Lett.

[CR13] Jain S, Chattopadhyay S, Jackeray R, Abid Z, Singh H (2016). Detection of *Salmonella typhi* utilizing bioconjugated fluorescent polymeric nanoparticles. J Nano Res.

[CR14] Joyner J, Wanless D, Sinigalliano CD, Lipp EK (2014). Use of quantitative real-time PCR for direct detection of *Serratia marcescens* in marine and other aquatic environments. App Environ Microbiol.

[CR15] Kasai H, Watanabe K, Gasteiger E, Bairoch A, Isono K, Yamamoto S, Harayama S (1998). Construction of the *gyrB* database for the identification and classification of bacteria. Genome Inform Workshop Genome Inform.

[CR16] Kunimoto D, Rennie R, Citron DM, Goldstein EJ (2004). Bacteriology of a bear bite wound to a human: case report. J Clin Microbiol.

[CR17] Mahon J, Lax AJ (1993). A quantitative polymerase chain reaction method for the detection in avian faeces of *Salmonellas* carrying the *spvR* gene. Epidemiol & Infect.

[CR18] Mann E, Hein I, Mester P, Stessl B, Rossmanith P, Wagner M, Dzieciol M (2013). A robust and poisson validated quantitative 5′ nuclease TaqMan® real-time PCR assay targeting *fimA* for the rapid detection of *Salmonella* spp. Food Food Anal Meth.

[CR19] Nam HM, Srinivasan V, Gillespie BE, Murinda SE, Oliver SP (2005). Application of SYBR green real-time PCR assay for specific detection of *Salmonella* spp. in dairy farm environmental samples. J Food Microbiol.

[CR20] Ngan GJ, Ng LM, Lin RT, Teo JW (2010). Development of a novel multiplex PCR for the detection and differentiation of *Salmonella enterica* serovars Typhi and Paratyphi A. Res Microbiol.

[CR21] Perelle S, Dilasser F, Malorny B, Grout J, Hoorfar J, Fach P (2004). Comparison of PCR-ELISA and LightCycler real-time PCR assays for detecting *Salmonella* spp. in milk and meat samples. Mol Cell Probes.

[CR22] Rd FJ, Davis BR, Hickmanbrenner FW, Mcwhorter A, Huntleycarter GP, Asbury MA, Riddle C, Wathengrady HG, Elias C, Fanning GR (1985). Biochemical identification of new species and biogroups of Enterobacteriaceae isolated from clinical specimens. J Clin Microbiol.

[CR23] Ruan JH, Wang WJ, Zhang TY, Bai QY, Zheng T, Zhang ZD, Wu LY, Huang YF, Yu DJ (2017). Rapid detection of *Serratia fonticola* by TaqMan quantitative real-time PCR using primers targeting the gyrB Gene. Curr Microbiol.

[CR24] Shanmugasundaram M, Radhika M, Murali HS, Batra HV (2009). Detection of *Salmonella enterica* serovar Typhimurium by selective amplification of *fliC*, *fljB*, *iroB*, *invA*, *rfbJ*, *STM2755*, *STM4497* genes by polymerase chain reaction in a monoplex and multiplex format. World J Microbiol Biot.

[CR25] Tasic S, Obradovic D, Tasic I (2013). Characterization of *Serratia fonticola*, an opportunistic pathogen isolated from drinking water. Arch Biol Sci.

[CR26] Tomás D, Rodrigo A, Hernández M, Ferrús MA (2009). Validation of real-time PCR and enzyme-linked fluorescent assay-based methods for detection of *Salmonella* spp. Chicken Feces Samples Food Anal Meth.

[CR27] Whelan JA, Russell NB, Whelan MA (2003). A method for the absolute quantification of cDNA using real-time PCR. J Immunol Methods.

[CR28] Yamamoto S, Harayama S (1996). Phylogenetic analysis of Acinetobacter strains based on the nucleotide sequences of *gyrB* genes and on the amino acid sequences of their products. Int J Syst Bacteriol.

[CR29] Zhu H, Sun SJ, Dang HY (2008). PCR detection of Serratia spp using primers targeting pfs and luxS genes involved in AI-2-dependent quorum sensing. Curr Microbiol.

