# Supplementary material for: Establishment of a duplex real-time qPCR method for detection of Salmonella spp. and Serratia fonticola in fishmeal
Source: AMB Express. 2020 Nov 24;10:207. doi: 10.1186/s13568-020-01144-x (PMC7686437; doi:10.1186/s13568-020-01144-x)
Supplement: Supplementary file 1 — Additional file 1: Table S1. Salmonella spp. strains used in this study. Table S2. Serratia genus strains used in this study. Table S3. Other strains used in this study. Table S4. Real-time qPCR primer pairs and probes used in this study. Table S5. Reproducibility and stability test of the duplex real-time PCR. Table S6. Parameters comparison between duplex real-time PCR and single real-time PCR. [file 13568_2020_1144_MOESM1_ESM.docx]

**Table S1** *Salmonella* spp. strains used in this study

| Bacterial species | Strain No. | No. of strains | Source of strains |
| --- | --- | --- | --- |
| *Salmonella enteritidis*  *Salmonella typhimurium*  *Salmonella montevideo*  *Salmonella weltevreden*  *Salmonella enteritidis*  *Salmonella birkenhead*  *salmonella anatis*  *Salmonella senftenberg*  *Salmonella lomita*  *Salmonella saintpaul*  *Salmonella* O:2  *Salmonella* O:4  *Salmonella* O:7  *Salmonella* O:8  *Salmonella* O:9  *Salmonella* O:3,10 | ATCC13076  GDM1.237  /  /  /  /  /  /  /  /  /  /  /  /  /  / | 1  1  2  1  3  1  1  1  1  1  3  1  2  2  3  5 | ATCC（Manassas, USA）  GDMCC(Guangdong, China)  Imported fishmeal  Imported fishmeal  Imported fishmeal  Imported fishmeal  Imported fishmeal  Imported fishmeal  Imported fishmeal  Imported fishmeal  Imported fishmeal  Imported fishmeal  Imported fishmeal  Imported fishmeal  Imported fishmeal  Imported fishmeal |

**Table S2** *Serratia* genus strains used in this study

| Bacterial species | Strain No. | No. of strains | Source of strains |
| --- | --- | --- | --- |
| *Serratia fonticola*  *Serratia marcescens*  *Serratia proteamaculans*  *Serratia odorifera*  *Serratia ficaria*  *Serratia plymuthica*  *Serratia rubidaea*  *Serratia liquefaciens*  *Serratia grimesii*  *Serratia fonticola* | GDM1.995  MCCC1A06806  MCCC1K00532  GDM1.864  GDM1.994  GDM1.996  GDM1.1008  CICC21538  ACCC01695  / | 1  1  1  1  1  1  1  1  1  3 | GDMCC (Guangdong, China)  MCCC (Xiamen, China)  MCCC (Xiamen, China)  GDMCC(Guangdong, China)  GDMCC (Guangdong, China)  GDMCC (Guangdong, China)  GDMCC (Guangdong, China)  CICC (Beijing, China)  ACCC(Beijing, China)  Imported fishmeal |

**Table S3** Other strains used in this study

| Bacterial species | Strain No. | No. of strains | Source of strains |
| --- | --- | --- | --- |
| *Eschericha coli*  *Citrobacter freundii*  *Shigella sonnei*  *Klebsiella oxytoca*  *Enterrococcus faecalis*  *Enterobacter aerogenes*  *Klebsiella pneumoniae* | ATCC25922  ATCC8090  ATCC25931  ATCC700324  ATCC29212  ATCC13048  20161226-16 | 1  1  1  1  1  1  1 | ATCC (Manassas, USA)  ATCC (Manassas, USA)  ATCC (Manassas, USA)  ATCC (Manassas, USA)  ATCC (Manassas, USA)  ATCC (Manassas, USA)  Imported fishmeal |

MCCC = Marine Culture Collection of China; GDMCC = Guangdong Microbial Culture Center; CICC = China Center of Industrial Culture Collection; ACCC = Agricultural Culture Collection of China

ATCC = [American Type Culture Collection](http://www.chazidian.com/dict/American%20Type%20Culture%20Collection/); GDMCC = Guangdong Microbial Culture Center

**Table S4** Real-time qPCR primer pairs and probes used in this study

| Primers and Probes | Length of product |
| --- | --- |
| *invA* -F: 5' -ATGGAAGCGCTCGCATTGTG-3'  *invA* -R: 5' -GGCTGAGGAAGGTACTGCCA-3'  *invA* -P:5' -JOE-TGCTCGTAATCCGCCGCCATTGGCG-BHQ1-3'  *gyrB* -F: 5' -TCGGTGAAACCGATCAGAC-3'  *gyrB* -R: 5' -GCCAGGATGTCGTACTCAAA-3'  *gyrB* -P:5' -6 FAM-CTGCGCTTCTGGCCGAGCTT-BHQ1-3' | 199bp    94bp |

**TABLE S5** Reproducibility and stability test of the duplex real-time PCR

| Tenfold dilution  (copies/μL） | |  | Ct value | | Ct _MN±SD_ | | CV（%） |
| --- | --- | --- | --- | --- | --- | --- | --- |
|  |  | 1 | 2 | 3 |  |  |  |
| 1.97×10^8^ | 19.989 | | 20.086 | 20.195 | | 20.090±0.103 | 0.51 |
| 1.97×10^7^ | 23.287 | | 23.331 | 23.302 | | 23.307±0.022 | 0.09 |
| 1.97×10^6^  1.97×10^5^  1.97×10^4^  1.45×10^8^  1.45×10^7^  1.45×10^6^  1.45×10^5^  1.45×10^4^ | 26.924  29.892  33.251  15.997  19.581  22.909  25.522  28.918 | | 26.851  29.934  33.525  16.005  19.330  22.724  29.526  29.209 | 30.134  33.342  15.856  19.343  19.343  22.898  25.628  29.146 | | 26.879±0.040  29.987±0.129  33.373±0.140  15.952±0.084  19.418±0.141  22.844±0.104  25.559±0.060  29.091±0.153 | 0.14  0.43  0.42  0.53  0.73  0.46  0.23  0.53 |

SD: Standard Deviation; MN: Mean; C.V (Coefficient of Variance) = (SD÷MN) × 100%

**Table S6** Parameters comparison between duplex real-time PCR and single real-time PCR

| Plasmid | Slope | | Y-axis intercept | | R^2^ | | Efficiency（%） | |
| --- | --- | --- | --- | --- | --- | --- | --- | --- |
|  | Single | Duplex | Single | Duplex | Single | Duplex | Single | Duplex |
| pUCm-*invA* | -3.267 | -3.362 | 48.116 | 48.775 | 0.998 | 0.999 | 102.344 | 98.346 |
| pUCm-*gyrB* | -3.242 | -3.409 | 42.919 | 44.396 | 0.999 | 0.999 | 103.429 | 96.49 |
