# Supplementary figures and images for: Establishment of a duplex real-time qPCR method for detection of Salmonella spp. and Serratia fonticola in fishmeal
Source: AMB Express. 2020 Nov 24;10:207. doi: 10.1186/s13568-020-01144-x (PMC7686437; doi:10.1186/s13568-020-01144-x)

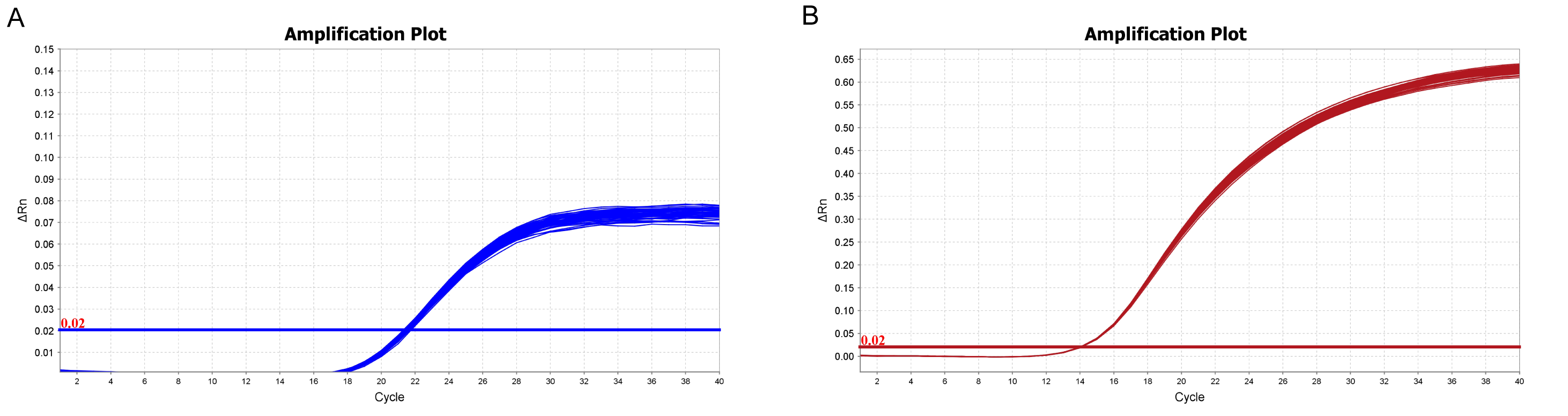

Supplement: Supplementary file 2 — Additional file 2: Fig. S1. Repeatability and stability test of RT-qPCR. 96 amplification plots for detection of Salmonella enteritidis (A) and S. fonticola (B). [file 13568_2020_1144_MOESM2_ESM.tif]
